# Comparing task performance, visual comfort and alertness under different lighting sources: an experimental study

**DOI:** 10.17179/excli2018-1676

**Published:** 2018-10-29

**Authors:** Reza Kazemi, Alireza Choobineh, Shirin Taheri, Pegah Rastipishe

**Affiliations:** 1 Research Center for Health Science, Institute of Health, Shiraz University of Medical Sciences, Shiraz, Iran; 2 Department of Ergonomics, School of Health, Shiraz University of Medical Sciences, Shiraz, Iran; 3 Student Research Committee, School of Health, Shiraz University of Medical Sciences, Shiraz, Iran

**Keywords:** lighting, performance, alertness, comfort

## Abstract

The aim of this study is to compare the effects of different light sources - namely light-emitting diode (LED), compact fluorescent (FLcomp) and fluorescent with warm color temperature (FLwarm) and cool color temperature (FLcool) - on the performances, alertness, visual comfort level and preferences in a pilot study. A laboratory controlled experiment was conducted by focusing on 20 postgraduate students who volunteered to participate in a series of tests under four different light sources. “GO NO GO” task and Karolinska Sleepiness Scale (KSS) were employed to assess objective and subjective alertness, while modified OLS questionnaire was used to gauge comfort level and preferences. In addition, editing and typing tasks were carried out as a performance evaluation. Significant increase was observed in subjective and objective alertness level under FLcool condition and LED in comparison to FLwarm and FLcomp (p < 0.05). In terms of typing performances, respondents performed significantly better with regard to typing speed under FLcool than FLwarm and FLcomp. The lowest number of typing errors was made under FLcool, followed by LED, FLcomp and FLwarm. LED was the most preferred (p=0.001) and most comfortable (p=0.011) lighting condition. The study concludes that the FLcool and LED were more beneficial for alertness level and performance for both computer-based and paper-based activities.

## Introduction

Over the past decades, different studies have shown that the features of workplace, especially lighting, can affect people's perception of mental fatigue, behavior and performance. These studies have shown that lighting considerably affects most of nonvisual performances, such as physiological and mental mechanisms, and cognitive-biological processes, such as circadian rhythms, alertness, core body temperature, hormone secretion and sleep, and is a known powerful and regulator factor of human circadian rhythms with environment (Samani and Samani, 2012[35]; De Kort and Smolders, 2010[9]; Borisuit et al., 2015[3]; Weinert et al., 2016[40]). Lighting exercises its impacts on body through the visual system. In the retina of human eye, there are light sensitive ganglion cells, which along with rod and cone receptors, are involved in the visual process and send light information to the circadian regulator and the suprachiasmatic nucleus of the hypothalamus in the brain via retinohypothalamic (Weinert et al., 2016[40]; Taufique and Kumar, 2016[37]). In this way, ganglion cells play a major role in coordinating the circadian rhythm and the cycle of darkness and light (Knez, 2014[20]; Sahin et al., 2014[33]). Studies show that light intensities and color temperature, as two important parameters in lighting, have different psychological and cognitive effects on humans (Sahin et al., 2014[33]). Many of these studies have examined the effect of light intensity on fatigue and sleep. Experimental studies have shown that exposure to higher levels of light at night leads to suppression of melatonin secretion, increase of physiological arousal, higher levels of mental consciousness and improvement of alertness and cognitive performance (Hawes et al., 2012[13]; Min et al., 2013[26]). Research findings have further demonstrated that increasing the color temperature of the light sources improves cognitive performance of night shift workers, visual performance and visual comfort of the students and sleep quality, performance and behavior of industrial staff (Viola et al., 2008[39]; Chellappa et al., 2011[7]; Shamsul et al., 2013[36]).

Nowadays, various types of lamps, including conventional fluorescent lamps, compact fluorescent lamps, incandescent bulb and LED lamps (manufactured after developing optimal consumption technologies) are used in residential and office environments. Although these lighting sources have different optical properties, few studies have compared these light sources in terms of their influence on people's performance, alertness and comfort. Therefore, it is unclear what type of light is more appropriate for office environments.

Comparing the effects of fluorescent lamps and halogens on performance, behavior and physical discomfort showed that there is no huge difference in performance of people exposed to these light sources in text editing, physical discomfort and behavior (Mayr et al., 2013[24]).

LED light is reported to be one of the best available sources in the market since it is polychromatic and most similar to daylight and has long lifespan, adjustable color temperature, and more light output per watt (Plitnick et al., 2010[31]). In a study, it was discovered that people who were exposed to LED enjoyed more visual comfort and color recognition than the ones who were in contact with fluorescent, with this effect being much more profound in higher color temperature. Considering the impact on attention and concentration, the best color temperature is reported to be 6500 K (Sahin et al., 2014[33]). 

In another study, it was demonstrated that LED lamps produce less mental fatigue than fluorescent lamps. It was also discovered that the average response time for performing spatial and verbal memory tasks while being exposed to fluorescent lamp is more than that of LED lamp (Canazei et al., 2017[6]). Research has also indicated that blue-rich white-light LED light source works better in activation of nonvisual performances in comparison with standard white fluorescent lamp (Okamoto and Nakagawa, 2015[28]).

Given that new LED and CFL light sources are being increasingly used in office environments, it is necessary to conduct a comprehensive study comparing these light sources and older ones (e.g. conventional fluorescent lamps) in terms of their effect on visual performance and visual comfort. Indeed, examining the effects of new light sources in office environments can be helpful for stakeholders to select the best one. As a result, the aim of this study is to compare these light sources on visual comfort, performance and alertness in an experimental study.

## Materials and Methods

### Subjects

This study adopted a repeated-measures experimental design. Twenty young and healthy postgraduate students (11 females and 9 males) were selected through purposive sampling from the Faculty of Health, Shiraz University of Medical Sciences (SUMS). The participants were right-handed and had normal or corrected visual acuity. The mean score for their age was 26.2 ± 1.6 years. The study was carried out during daytime over a 2-month period ranging from October, 1, 2017 through November, 30, 2017. The research was designed in such a way that each subject has four experimental days (with one 2 hours trial per four lighting conditions). The timing for performing the test on every day was approximately similar for every subject.

### Setup of experiment

The experiment was carried out in a confined room which had no windows or external source of light, with dimensions of 3 m ×1.5 m × 2.6 m. The room was located in the occupational health laboratory of School of Health, SUMS.

**Figure 1:**
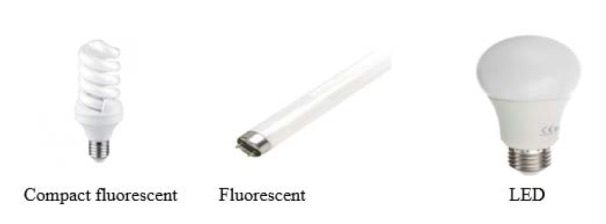
The lamps used in the study

The lighting sources included conventional fluorescent, compact fluorescent and LED lamps with various color temperatures (Figure 1(Fig. 1)). The illumination level of the studied environment was adjusted to 300 ± 10 lux for every four lamps. Table 1(Tab. 1) displays the features of the used light sources.

### Instruments 

#### Subjective alertness

Karolinska Sleepiness Scale (KSS) was employed to assess variations in the participants' alertness. This scale, which has already been validated against EEG data by Åkerstedt and Gillberg (1990[1]), is a subjective rating in which each person indicates their current alertness level on a 9-point likert scale ranging from (1) “extremely alert” to (9) “fighting sleep”. Various studies have utilized KSS to examine drowsiness and consciousness in different workplaces (Kazemi et al., 2016[16], 2018[17][18]).

#### Objective alertness

The go/no go task is an objective measure of cognitive alertness (Heath et al., 2014[14]). The 4-min computerized task displayed a series of single black letters (either ''M'' or ''W'') on a white background using an Acer laptop. Each letter was displayed for 0.216 sec and the blank inter-trial interval time varied randomly between 1300 and 1700 ms. The participants responded only to the letter ''W'' by pressing the spacebar. The response time window was between 150 ms (to stop anticipated responses) and 1500 ms. After 500 ms, a 440-Hz tone sounded for 475 ms to encourage the participants to respond. The letter ''W'' appeared randomly at a frequency of 4 in every 20 letters displayed. Average reaction time (ms), and accuracy responses (%) were recorded.

### Performance at computer-based task

A computer-based task was developed to examine the effect of different lighting conditions on the subjects' performance. That is, each subject was required to type a Persian article using Microsoft Office Word 2007 by referring to the hard copy in 10 minutes. Overall, three different articles (one article for each lighting condition), each of which was around 400 words long, were presented to the participants for typing. The subjects' typing performance test was assessed in the light of typing speed (total number of words typed) and typing accuracy (percentage of typos) in every environment (lamp type). It should be noted that the automatic spelling and grammar check were disabled before the participants began the test.

### Performance at paper-based task

In addition to the computer-based task, a paper-based task was employed to further explore the impact of the three lighting conditions on respondents' alertness. This task entailed proofreading 3 Persian articles, with each one being 600-620 words long. Each article was printed on two separate A4 sheet papers and the participants were given 3 minutes to proofread each text. B Nazanin font type (size 14) was used for typing the texts, hence all three passages consisted of 34-36 lines. Each text contained 15 spelling errors (letter omissions, letter substitutions, transpositions of adjacent letters and letter additions) and 10 syntactic ones (e.g. incorrect flection and conjugation), which were randomly scattered in the passage. Indeed, the presence of syntactic errors guaranteed that the participants would read the text for understanding rather than simply scanning it for detecting spelling mistakes. The participants' performance accuracy was gauged through dividing the number of correctly detected errors by the number of false alarms. Further, their proofreading speed was assessed based on the number of lines read within the reading time.

### Visual comfort and subjective preferences assessment

A modified version of Office Lighting Survey (OLS) was utilized to assess the respondents' satisfaction with each lighting environment (Shamsul et al., 2013[36]). The questionnaire was composed of both general and artificial lighting-specific statements. The respondents should indicate the degree of their agreement with each statement on a 4-point scale (yes, rather yes, rather no, no). Thus, the survey did not have any neutral option.

### Statistical analysis

The collected data were analyzed by Statistical Package for the Social Sciences (SPSS) 21 (SPSS Inc., Chicago, IL, USA). The Kolmogorov-Smirnov test was used to assess the normality of data distribution. The participants' objective and subjective alertness before and after exercise in the four experiments were compared using a series of paired samples t-tests. The effect of lighting source on all measurements was tested by a repeated measures analysis of variance (ANOVA) for each of the dependent variables to determine if there were any significant differences before and after the experiment. Then, LSD tests were conducted as the follow-up posthoc to see where the significant differences lied. The statistical significance was set at 0.05.

### Study procedures

At the beginning of the study, the subjects were given a written consent form to sign. They were informed that participation in the study was voluntary and there would be no repercussions if they decided not to take part in it. Upon signing the consent form, the participating students entered the experiment.

**Figure 2:**
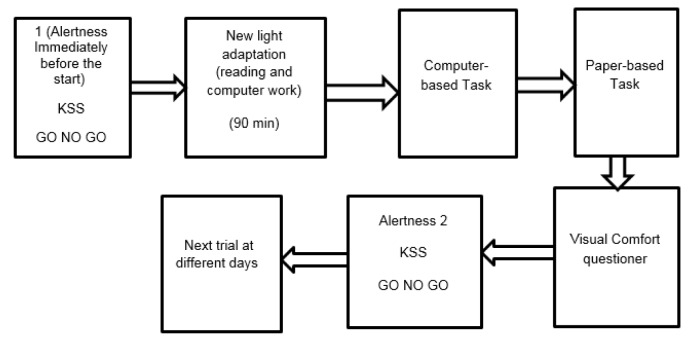
Flow of procedures of our experiment

In the experimental phase, the sequence of lighting conditions was randomized, with each trial being approximately completed in about 2 h (Figure 2(Fig. 2)). The participants were invited to complete KSS and “GO NO GO” task prior to being exposed to the lighting conditions. Then, their exposure to different lighting environments lasted for 15 minutes, followed by completing the paper-based task.

## Results

### Subjective alertness

**Figure 3:**
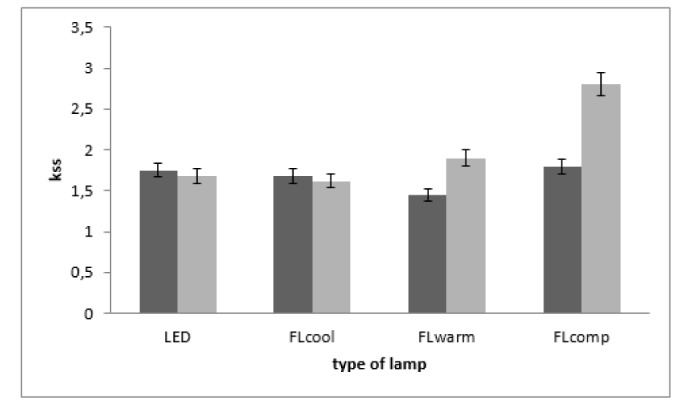
Comparison of alertness level (mean) before and after the trial between light sources

The impact of different lighting conditions on the participants' subjective alertness level is displayed in Figure 3(Fig. 3). No significant discrepancy was observed in the alertness level of the participants under different lighting conditions in the pre-experiment stage. Comparing pre- and post-experiment alertness levels revealed a significant decline in FLcomp and FLwarm (p < 0.05). In contrast, alertness level measurably went up under FLcool and LED conditions, though no significant difference was detected between these two conditions in the post-experiment phase (p > 0.05).

The results of repeated measures analyses of variance revealed significant differences in alertness [F (3, 57) = 7.5; p < 0.001] among the four experimental conditions. The LSD post hoc test was used for pairwise comparisons to assess alertness during four experimental conditions. The respondents' score in the FLcomp environment was found to be considerably higher than that of the LED (p < 0.05) and FLcool environments (p < 0.05). Thus, the participants were more alert under the FLcool and LED conditions.

### Objective alertness

**Figure 4:**
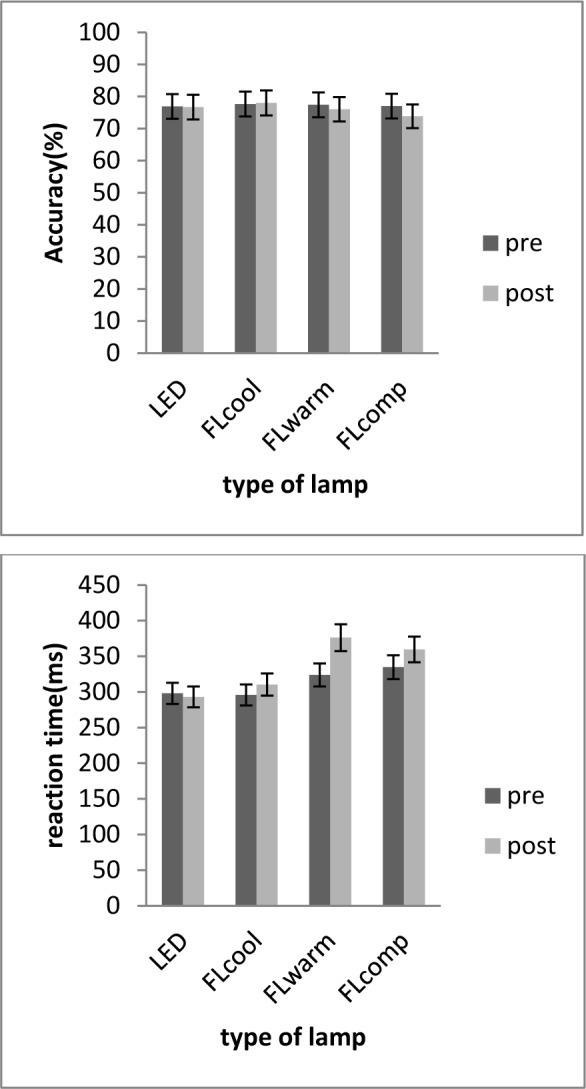
Mean percentage accuracy (left panel) and reaction time (right panel) on the GO/NOGO task over four lighting condition. Error bars are standard errors.

The results presented in Figure 4(Fig. 4) show “GO NO GO” task accuracy (%) and reaction time (ms) in four environments with different lightings. The results of paired samples t-tests demonstrated that, considering the FLcool condition, a significant decline was observed in the post-experiment accuracy (%) (p < 0.05) (Figure 4(Fig. 4)). In contrast, compared to the pre-experiment phase, “GO NO GO” reaction time in post experiment increased significantly under FLwarm condition (p < 0.05) (Figure 4(Fig. 4)). There was no significant difference between the pre and post LEDs and FLcool condition regarding the reaction time and the accuracy of “GO NO GO” task.

The results of repeated measures analyses of variance revealed significant differences in reaction time [F (3, 57) = 7.5; p < 0.001] and accuracy [F (2.7, 57.8) = 3.5; p < 0.024] of “GO NO GO” task among the four experimental conditions. The LSD post hoc test was used for pairwise comparisons to assess GO NO GO scores during the four experimental conditions. Significant difference in accuracy (%) was found between LED and FLcomp (P = 0.01). In addition, there was a significant difference in reaction time between LED and FLcomp (P = 0.03), LED and FLwarm (P = 0.04), as well as between FLcool and FLcomp (P = 0.03).

### Performance at computer-based task

**Figure 5:**
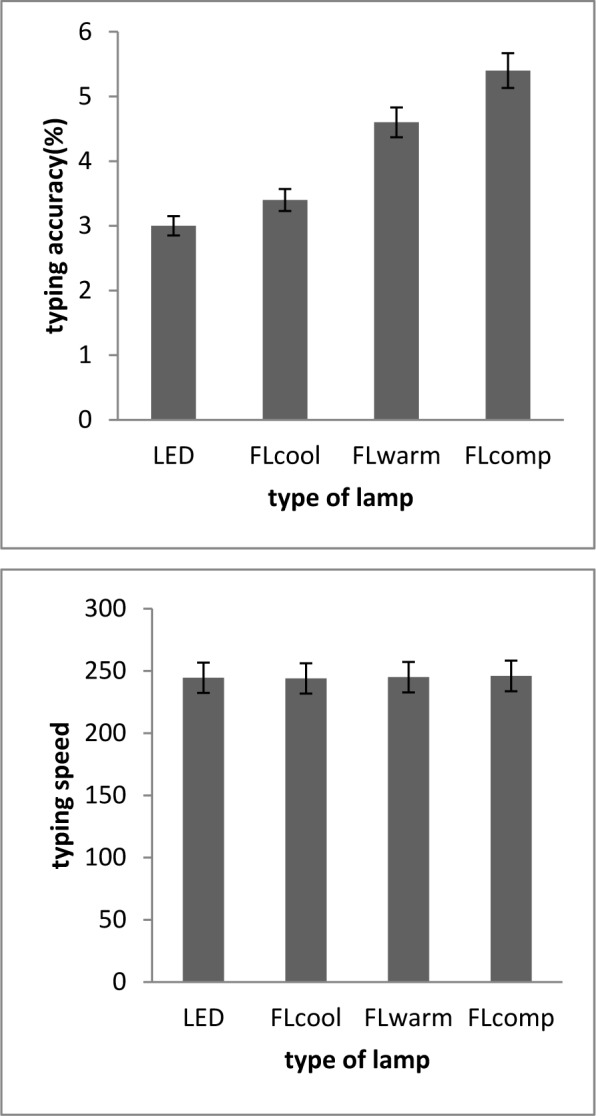
Comparison of typing performances of subjects in four different lighting conditions

The results presented in Figure 5(Fig. 5) show typing performance, including speed and accuracy, in the four environments with different lightings. Analysis of variance of repeated data showed that there is a significant difference in typing speed between different situations [F (2, 36) = 7.00; p < 0.05].

Further analysis with paired comparison analysis of environmental conditions showed that, under fluorescent lighting with high color temperature (6500), typing is performed with higher speed in comparison FLcool (3500 k) (P= 0.02) and FLcomp lighting (P= 0.04). In contrast, no significant difference was seen in the typing speed while the participants were exposed to the other lamps.

Also, analysis of variance of repeated data showed that there is a huge difference in typing accuracy among the studied people in different lighting conditions [F (2.5,44) = 17.2; p =0.001]. Paired comparison of lighting condition showed that typing accuracy in lighting with FLcool of 6500 K is higher than that among participants exposed to FLwarm (p=0.002) and FLcomp lamp (p=0.001). Also, typing accuracy in lighting with LED is significantly higher than that under the conditions of FLcool and FLcomp lamp with (p=0.02) and (p=0.001) respectively. No significant difference was observed in the typing accuracy of participants exposed to the LED lamp and the FLcool.

### Performance at paper-based task

**Figure 6:**
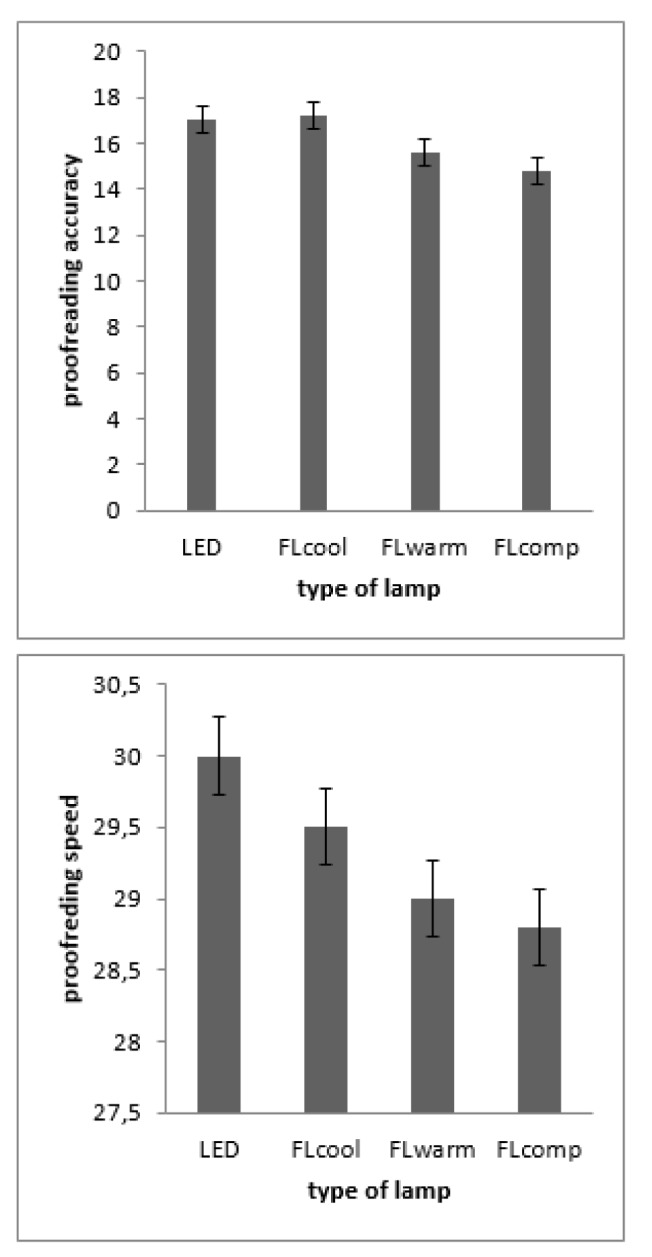
Performance in the proofreading task as a function of light source. The left panel depicts proofreading accuracy measured as the average number of correctly detected errors corrected by the number of false alarms. The right panel depicts proofreading speed measured as the average number of lines read. The error bars depict the standard errors of the means.

Edition accuracy and speed as a paper-based task is shown in Figure 6(Fig. 6). No significant difference was seen among different lighting conditions in edition accuracy [F (2.00, 57.30) = 1.40, p > 0.05]. Nonetheless, there are significant differences among different lighting conditions in edition speed [F (1.8, 35.50) = 7.80, p < 0.05]. Paired comparison revealed that edition speed in lighting with FLcool is significantly better than that in FLwarm (p=0.05) and FLcomp (p=0.02). On the other hand, edition speed in lighting with LED is significantly better than that among participants exposed to FLcomp (p=0.03). No significant difference was seen between edition speed in lighting with LED and FLcool. 

### Subjective preferences and visual comfort

**Figure 7:**
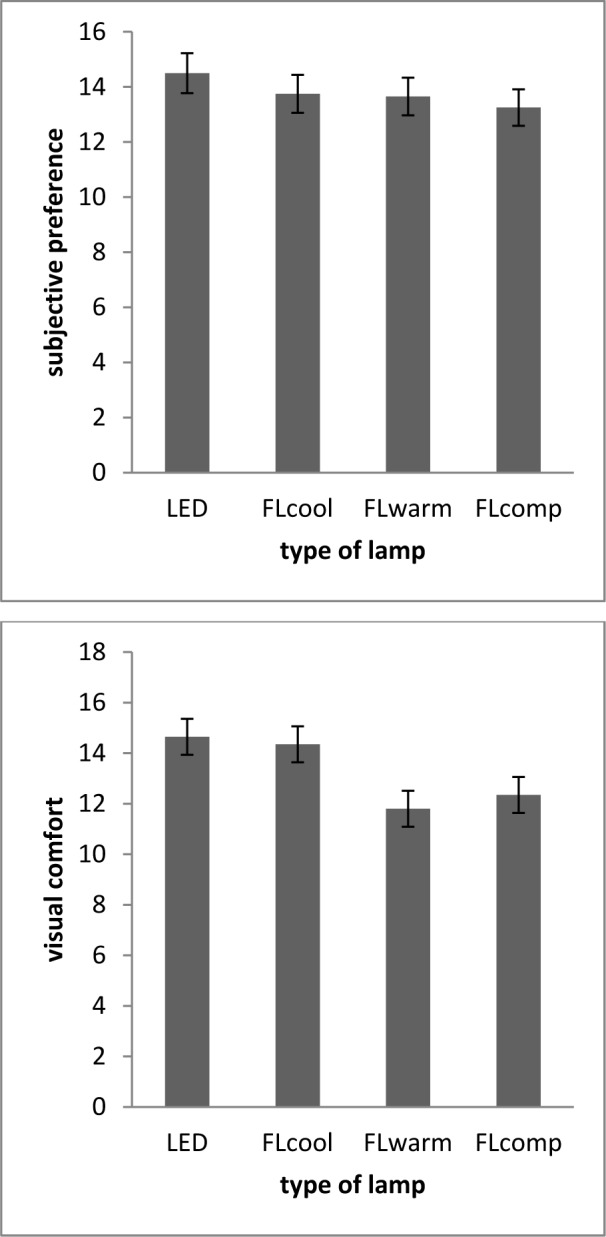
Comparison of subjective preference (left panel) and visual comfort level (right panel) of participants for three different lights.

The results showed that there are significant differences among preference usage under the three lamp conditions (Figure 7(Fig. 7)) [F (2.5, 46) = 19.80, p < 0.05]. Paired comparison showed that the participants preferred LED lamp to FLcool (p=0.008), FLwarm (p=0.001) and FLcomp (p=0.001). However, no significant difference was seen in participants' preferences for FLcool, FLwarm in this range. Also, significantly disparate visual comfort scores were reported by the participants while working under different lighting conditions (Figure 7(Fig. 7)) [F (2.5, 46) = 19.80, p < 0.05]. In particular, the participants reported more visual comfort in using LED lamps and FLcool. Paired comparison showed that visual comfort of LED is higher than that of FLcomp and FLcool (p=0.01, p=0.001 respectively). Also, FLcool is reported to be better than FLcomp (p=0.004), although visual comfort in working with FLcool was more suitable (p=0.12).

## Discussion

Lighting is an important factor in improving employees' performance, health, safety and efficiency in work environment (Dianat et al., 2013[10]). However, little attention has been paid to lighting sources, especially new ones, in office environment where employees have to work long hours while being exposed to artificial lighting. There are various types of lighting sources with different characteristics especially in terms of their appearance, operating mechanism and color temperature. These features make it hard to select the most suitable one with respect to their effect on human efficiency, alertness and visual comfort. Some studies have investigated performance (especially mental performance) while using different lighting sources. However, the current study adopted a new outlook in two areas. First, this study was done as a pilot with more real tests such as typing and editing, which are examples of office employees' tasks that were absent in other studies. Second, it investigated the effect of type and color temperature of lamps. To this end, the study focused on the effects of three different types of lamps (i.e. LED lamp, FL and FLcomp) with two color temperatures of 3500 K and 600 K. Of particular importance was investigating the effect of type of lamp and color temperature on employees' performance, alertness and visual comfort in simulated office duties.

The results showed that, for most of the investigated indicators, the LED lamp and FLcool were better than the other two (3500 K). On the other hand, investigated indicators for FLcool and FLwarm were significantly different. Hence, it can be concluded that the color temperature (rather than the lamp type) is the main factor causing difference among performance, visual comfort and satisfaction. Therefore, the color temperature of 6500 K is recognized to be the best for office duties. 

Performance of computer and traditional tasks while using LED lamp was better than that when using FLwarm and FLcomp. This result is in accordance with other studies' findings (Reyes et al., 2009[32]). Sahin and co-workers (2014[33]) found that LED lamp with color temperature of 6500 K is more effective in terms of its impact on attention and concentration. The same results were reported by Shamsul et al. (2013[36]), who found that students' performance in speed and accuracy of typing significantly improve using lighting with high color temperature in comparison with low color temperature (Shamsul et al., 2013[36]). To justify this result, it could be mentioned that by increasing the color temperature of lighting sources, a rise is observed in the percentage of blue spectrum, which is recognized to be an important factor in performance and alertness improvement. In studies done by other researchers, color temperature has also been investigated as an effective factor. As an example Ferlazzo et al. (2014[11]) showed that exposure to cold light improves the capacity of cognitive system in performing complex tasks. Also, Motamedzadeh et al. (2017[27]) and Baek and Min (2015[2]) studying the effect of light color temperature on cognitive performance of night shift workers, displayed that lighting sources with higher color temperature have a better effect on peoples' cognitive performance than lighting sources with lower color temperature.

These impacts can also be attributed to the nonvisual effects of light. Different studies have investigated nonvisual effects and mechanism of light. Light improves cognitive performance via two mechanisms. Kretschmer et al. (2012[21]) showed that bright light improves working memory and concentration by exiting the sympathetic nervous system. On the other hand, reducing the level of melatonin hormone and increasing alertness due to high sensitivity of circadian rhythms to blue light can be recognized as an effective factor in cognitive performance improvement. Also, Baek and Min (2015[2]) have demonstrated that blue light reduces the brain alpha wave activity and helps the cognitive performance in sustained attention after having lunch, which is in accordance with this study's results. Klimesch stated that reduction of brain alpha and theta wave improves working memory process and attention (Klimesch, 1999[19]). It has also been indicated that the blue light can significantly improve brain activities in the cerebral cortex, which includes working memory and executive control (Cabeza and Nyberg, 2000[4]). Vandewalle and others assumed that the blue light can ease the healing buffer of attention deficit, hence improving the cognitive performance.

The other investigated factor in this study was alertness, which was studied via subjective and objective measures. The results showed that, like performance enhancement, color temperature is an important factor for improving alertness, whereas no significant difference was seen between LED lamps and fluorescent with roughly the same color temperature lamps. In fact, by increasing the temperature, the richness of blue light will also increase and alertness will improve. Numerous studies have been conducted concerning the effect of intensity of light on alertness, with their results corroborating the findings of the present study (Czeisler and Dijk, 1995[8]; Hoffmann et al., 2008[15]; Lowden et al., 2004[22]; Cajochen et al., 2014[5]). This outcome might be due to brain activities and human auto nerve system improvement because of an increase in percentage of the blue light in lighting source with high color temperature. 

Phipps-Nelson et al. (2009[30]) showed that light can excite alertness in human. A study carried out in laboratory with 50 minutes exposure to 470 nm light (blue light) showed that the blue light can increase alertness level (Phipps-Nelson et al., 2009[30]). As a result of an experimental study, Phipps-Nelson et al. (2009[30]) found that blue light stimulates brain delta and theta wave activities and thus increases alertness. The results of this study are also in line with those obtained by Viola et al. (2010[39]). Exposing workers of different shift schedules to lighting sources with different color temperature during 4 weeks, they showed peoples' alertness and sleep quality improve by increasing color temperature of lighting sources (Viola et al., 2010[39]).

The third factor which was investigated in this study was subjective preferences and visual comfort. Although no significant difference was observed in subjective preferences among the four lighting conditions, the participants preferred using LED lamps over fluorescent and compact fluorescent lamps. The results of this study are not in accordance with other studies in this area. In the study by Shamsul, the studied people preferred lighting of the lamps with color temperature of 4000 K over light with color temperature of 3000 K and 6500 K (Shamsul et al., 2013[36]). Also, Park et al. (2010[29]) found that 55 % of men and 24 % of women preferred light with color temperature of 4000 K over light with color temperature of 3000 K and 5000 K. On the other hand, researchers have reported different personal preferences. For example, Miller (2012[25]) showed that, at daylight intensity of 500 lux, the average preferred color temperature is 3300 K, while Samani (2011[34]) showed that people typically prefer warmer color temperature. It seems that preference for CCT of light is strongly mental and a person may prefer different color temperatures during different hours of the day.

Concerning visual comfort, this study showed that there was no significant difference among three types of LED light, fluorescent with warm temperature and compact fluorescent, but a significantly better visual comfort was reported while the participants were exposed to FLcool. These results are in contrast with the ones obtained in other studies. In a study done by Shamsul, the participants reported more comfort while being exposed to higher color temperature (Shamsul et al., 2013[36]). Also, Manva showed that people feel more comfortable while being exposed to color temperature of 400 K than 2700 K in office environment (Manav, 2007[23]). Nonetheless, a study done by Park et al. (2010[29]) showed that people preferred color temperature of 3000 K over higher color temperature in terms of comfort. Additionally, Górnicka showed that color temperature of 17000 K causes dizziness and is unpleasant to people (Górnicka, 2008[12]). 

### Limitations

Like any other research, the current study suffers from some limitations. First, only university students were included in the study, meaning that caution should be exercised in generalizing the findings to other groups of people. Additionally, since a self-report instrument was implemented to collect the data, the findings might be biased and reliability might be negatively influenced. Besides, it was impossible to adopt a double-blinded design in this study. Thus, the obtained results may be inevitably influenced by Howthorne effect. That is, the participants might have performed significantly better (or worse) than the normal condition given that they knew they were being studied.

## Conclusion

The results of this study showed that human performance was better under exposure to cold color temperature than warm and semi warm color temperature. On the other hand, the participants demonstrated significantly better performance in working with fluorescent and LED lamps with the same color temperature (6500k) than other lighting sources. Of course, a significant difference was detected in performances while the participants were exposed to fluorescent with high (6500 k) and low (3500k) color temperature, indicating that performance is affected by color temperature not lamp type. On the other hand, the participating students did not have any preference in using studied lamps but they felt more comfort using lamps with cold color temperature. Thus, choosing lighting sources with appropriate color temperature and considering peoples' opinion in selecting lamp is recommended to engineers and designers in order to improve performance, lighting quality and workers' satisfaction. Comprehensive and longitudinal studies on different available types of lamps should be done to be able to draw firm conclusions. Also, using new and more accurate methods for evaluation is recommended for future studies. 

## Acknowledgement

Research funding for this study was provided by Research center of health science, Shiraz University of Medical Sciences, via project no. 95-01-42-13630.

## Conflict of interest

The authors declare no conflict of interest.

## Figures and Tables

**Table 1:**
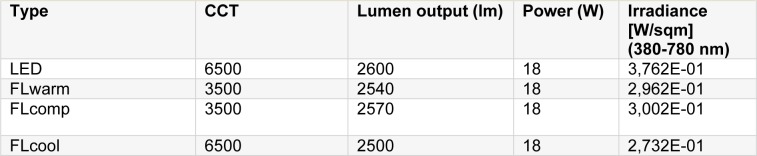
Physical properties of lamps
